# Eggs early in complementary feeding increase choline pathway biomarkers and DHA: a randomized controlled trial in Ecuador

**DOI:** 10.3945/ajcn.117.160515

**Published:** 2017-11-01

**Authors:** Lora L Iannotti, Chessa K Lutter, William F Waters, Carlos Andres Gallegos Riofrío, Carla Malo, Gregory Reinhart, Ana Palacios, Celia Karp, Melissa Chapnick, Katherine Cox, Santiago Aguirre, Luis Narvaez, Fernando López, Rohini Sidhu, Pamela Kell, Xuntian Jiang, Hideji Fujiwara, Daniel S Ory, Rebecca Young, Christine P Stewart

**Affiliations:** 1 Brown School, Institute for Public Health, and; 2 Diabetic Cardiovascular Disease Center, Washington University in St. Louis, St. Louis, MO;; 3 School of Public Health, University of Maryland, College Park, MD;; 4 RTI International, Research Triangle Park, NC;; 5 Institute for Research in Health and Nutrition, Universidad San Francisco de Quito, Quito, Ecuador;; 6 The Mathile Institute for the Advancement of Human Nutrition, Dayton, OH;; 7 Department of Nutritional Sciences, The University of Texas at Austin, Austin, TX;; 8 Johns Hopkins Bloomberg School of Public Health, Baltimore, MD;; 9 NETLAB Laboratorios Especializados, Quito, Ecuador; and; 10 Department of Nutrition, University of California, Davis, Davis, CA

**Keywords:** egg nutrition, children, choline, betaine, vitamin B-12, docosahexaenoic acid

## Abstract

**Background:** Choline status has been associated with stunting among young children. Findings from this study showed that an egg intervention improved linear growth by a length-for-age *z* score of 0.63.

**Objective:** We aimed to test the efficacy of eggs introduced early in complementary feeding on plasma concentrations of biomarkers in choline pathways, vitamins B-12 and A, and essential fatty acids.

**Design:** A randomized controlled trial, the Lulun (“egg” in Kichwa) Project, was conducted in a rural indigenous population of Ecuador. Infants aged 6–9 mo were randomly assigned to treatment (1 egg/d for 6 mo; *n* = 80) and control (no intervention; *n* = 83) groups. Socioeconomic data, anthropometric measures, and blood samples were collected at baseline and endline. Household visits were made weekly for morbidity surveillance. We tested vitamin B-12 plasma concentrations by using chemiluminescent competitive immunoassay and plasma concentrations of choline, betaine, dimethylglycine, retinol, essential fatty acids, methionine, dimethylamine (DMA), trimethylamine, and trimethylamine-*N*-oxide (TMAO) with the use of liquid chromatography–tandem mass spectrometry.

**Results:** Socioeconomic factors and biomarker concentrations were comparable at baseline. Of infants, 11.4% were vitamin B-12 deficient and 31.7% marginally deficient at baseline. In adjusted generalized linear regression modeling, the egg intervention increased plasma concentrations compared with control by the following effect sizes: choline, 0.35 (95% CI: 0.12, 0.57); betaine, 0.29 (95% CI: 0.01, 0.58); methionine, 0.31 (95% CI: 0.03, 0.60); docosahexaenoic acid, 0.43 (95% CI: 0.13, 0.73); DMA, 0.37 (95% CI: 0.37, 0.69); and TMAO, 0.33 (95% CI: 0.08, 0.58). No significant group differences were found for vitamin B-12, retinol, linoleic acid (LA), α-linolenic acid (ALA), or ratios of betaine to choline and LA to ALA.

**Conclusion:** The findings supported our hypothesis that early introduction of eggs significantly improved choline and other markers in its methyl group metabolism pathway. This trial was registered at clinicaltrials.gov as NCT02446873.

## INTRODUCTION

Multiple biological pathways influence early growth and development and depend on the adequate intake and metabolism of essential nutrients during infancy and early childhood. Choline is necessary for the production of phospholipids, cell membrane integrity, and in the conversion of acetylcholine and sphingomyelin in brain development and function. Alternatively, choline may be converted to betaine, which plays a crucial role as a methyl donor, converting homocysteine to methionine in remethylation pathways. This leads to the production of *S*-adenosylmethionine, enabling substrate methylation (DNA, RNA, proteins, and lipids) (1). In the gut, choline may be converted by the gut microbes into trimethylamine (TMA), which is then readily absorbed and converted into trimethylamine-*N*-oxide (TMAO) in the liver (2). TMAO has been implicated as a cardiovascular disease risk factor in adults, although its importance in infant and child health is unknown (3).

Like milk or seeds, eggs holistically support the early growth and development of an organism and are, therefore, dense in nutrient content. They are among the richest sources of choline and other important macro- and micronutrients necessary for growth (4, 5). Similar to other animal-source foods, eggs provide vitamin B-12 and have been shown to provide essential fatty acids including DHA (22:6n–3) (6–8). Current evidence, primarily from upper-income countries, shows that eggs introduced during infancy neither increase allergy incidence risk or egg sensitization (9–11). We hypothesized that by introducing eggs early in the complementary feeding period in a setting of high background stunting there would be important growth and nutrition benefits.

The Lulun Project, a randomized controlled trial (RCT) conducted in a mixed-indigenous rural Andean community of Ecuador, aimed to test the efficacy of eggs given early in the complementary feeding period on improved nutrition biomarkers and growth outcomes. We recently reported findings from a study that showed that eggs increased linear growth by a length-for-age *z* score of 0.63 (95% CI: 0.38, 0.88) and reduced stunting by 47% (prevalence ratio: 0.53; 95% CI: 0.37, 0.77) (12). We hypothesized that the intervention of 1 egg/d for 6 mo would significantly increase choline, vitamin B-12, and essential fatty acid biomarkers.

## METHODS

### Study design and participants

Details of the study design and methods are described elsewhere (12). In brief, this was an RCT with a parallel design and a 1:1 ratio of intervention to controls. The study was conducted in Cotopaxi Province, Ecuador, a rural region with ∼457,000 residents, of whom 22% identified as indigenous in the last census (13). Mother (caregiver)-infant pairs were recruited over a 5-mo period by a field team from Universidad San Francisco de Quito (USFQ). Eligibility criteria were as follows: infant aged 6–9 mo, singleton birth, and healthy without fever, a congenital heart condition, severe malnutrition, or egg allergy. Written informed consent was obtained from mothers or caregivers before baseline data collection.

Ethics approval was obtained from participating institutions, including USFQ, Washington University in St. Louis, and the Pan American Health Organization in Washington, DC. The trial is registered at clinicaltrials.gov as NCT02446873.

### Randomization, procedures, and intervention

Community meetings were first held to discuss the Lulun (“egg” in Kichwa) Project and begin recruitment. Groups of 8–10 potentially eligible mother (caregiver)-pairs were then gathered at temporary data collection points. After the informed consent process, the pairs were randomly assigned to the intervention or control groups. Mothers or other caregivers were shown the forms, labeled “alpha” and “beta,” which were then hidden and sealed and placed in a container. Each participant then drew a form for group assignment. The egg intervention precluded blinding participants and study enrollment and surveillance teams, who monitored participants in both groups throughout the trial for morbidities. Data collection staff, who measured child anthropometric variables and dietary intake, laboratory staff, and data analysts were blinded to group assignment.

The intervention consisted of 1 egg/d for a 6-mo period, delivered weekly in batches of 7 eggs. Weekly surveillance visits were made to both the intervention and control groups to collect information about morbidities and to make referrals to local health care facilities in the case of a seriously ill child. For the intervention group, surveillance team members also recorded information in a log report about egg consumption by the child and any complications associated with egg consumption. A 24-h frequency of dietary intake measure, including eggs, was conducted in both groups at baseline and endline. Eggs were reported to be consumed in the control group for 45% and 60% of children and in the intervention group for 40% and 91% of children at baseline and endline, respectively (12). Social marketing activities, including messaging at data collection sites, workshops, and sporting and entertainment activities, were directed to all participants to encourage participation and compliance.

Eggs were procured from 5 small-to-medium-size poultry farms in the surrounding areas. The poultry feeds contained corn, soy, wheat bran, rice powder, palm oil, and molasses, among other ingredients with comparable nutrient content. The nutrient content of a sample of 9 eggs sourced from 1 study supplier was tested by Eurofins Scientific Nutrition Analysis Center (Des Moines, Iowa) in April 2016. The eggs were hard-boiled and refrigerated before being transported to the United States for testing. Elemental and nutritional testing was completed on eggs and focused on those being examined as biomarkers in the children, with additional nutrients added beyond standard profiles.

### Primary outcomes

The primary outcomes were changes in plasma concentrations of nutrient biomarkers, including choline, betaine, dimethylglycine (DMG), and linoleic acid (LA; 18:2n–6), α-linolenic acid (ALA; 18:3n–3), and DHA, and child-growth anthropometric measures. These outcome measures were collected at baseline and at endline follow-up at 6 mo. Anthropometric measures of length and weight and conversion to *z* scores are described elsewhere (12). Surveys were also administered to mothers (caregivers) for socioeconomic, demographic, and child dietary intake and morbidity information.

NETLAB, a reference laboratory located in Quito, Ecuador, was responsible for collecting, processing, and storing the blood samples during the trial and for the vitamin B-12 analyses. This facility regularly collaborates with USFQ and was responsible for all nutrient biomarkers in a recent national nutrition survey, Encuesta Nacional de Salud y Nutrición (ENSANUT-ECU) 2012 (14). Trained phlebotomists from NETLAB accompanied the Lulun study team to data collection sites. A total of 3 mL of blood was collected from each child. Blood was separated into aliquots at the field site into different tubes containing different anticoagulants based on the intended assays: lithium heparin for the choline markers and fatty acids and EDTA for vitamin B-12. Tubes were shielded from light, placed on ice, and transported back to the NETLAB facility in Quito. Samples were centrifuged at 500 × *g* for 10 min at 4°C; vials with plasma were then stored in a NETLAB freezer at −80°C. At the close of the study, the samples intended for analyses of choline-related markers and fatty acids for were packed in dry ice and shipped to the Metabolomics Laboratory at Washington University in St. Louis.

### Laboratory methods

Vitamin B-12 plasma concentrations were assessed by using chemiluminescent competitive immunoassay (IMMULITE 1000 Analyzer; Diagnostic Products). Both internal and external quality controls were carried out. When values were outside acceptable ranges, we conducted duplicates. The CV of the internal control was 8.14% and total error was 20.9%, both in range of minimal biological variation (11.3% and 45.04%, respectively) during the run of all the samples.

For the choline, betaine, DMG, methionine, dimethylamine (DMA), TMA, TMAO, retinol, and fatty acid measures, we used modified liquid chromatography–tandem mass spectrometry (LC-MS/MS) methods (15–19). Sample analysis was performed with a 20AD HPLC system (Shimadzu), coupled to a triple quadrupole mass spectrometer (4000 QTRAP; AB Sciex) operated in multiple reaction monitoring mode. The positive ion electrospray ionization mode was used. Retinol and DMA were analyzed on an API 4000 mass spectrometer (AB Sciex) coupled with a Prominence LC-20AD HPLC system (Shimadzu). For details about instrument parameters for LC-MS/MS analysis of biomarkers and their internal standards, see **Supplemental Table 1**.

Protein precipitation was first performed to extract choline, betaine, DMG, free methionine, fatty acids, TMAO, TMA, and DMA from human plasma. Deuterated d_3_-betaine, d_9_-choline, d_6_-DMG, d_3_-Met, d_4_-LA, d_5_-ALA, d_5_-DHA, d_9_-TMAO, d_6_-DMA, and d_6_-retinol were used as internal standards and were added to the samples before extraction. The resulting extracts were divided into 2 parts. One part was used for the analysis of choline, betaine, and DMG. The other part was further processed to the amino-methylphenylpyridium derivative to increase the mass spectrometry sensitivity and selectivity of LA, ALA, and DHA. The 6- to 11-point calibration standards of all of the analytes containing respective deuterated internal standards were also prepared for absolute quantification. For the DMA assay, the samples were derivatized with pyridine-3-sulfonyl chloride to improve detection sensitivity. The TMA in the samples was oxidized to TMAO with meta-chloroperoxybenzoic acid and calculated from pre- and postoxidized TMAO values. Data processing was conducted with Analyst 1.5.1 (Applied Biosystems).

### Statistical analysis

Sample size was estimated on the basis of hypothesized change (difference-in-difference with control) in plasma vitamin B-12 concentration of 59.4 pmol/L over the 6-mo intervention period and on other previous findings of vitamin B-12 supplementation and the variability in vitamin B-12 status among infants (20, 21). This nutrient was selected for sample size calculation because sufficient data were available to estimate variability and potential effects from egg consumption in contrast to the other nutrients studied. Assuming a 20% attrition rate, we estimated the requirement of 90 children/group with the use of estimates for 2 samples with repeated measures (α = 0.05, 1 − β = 0.80).

Intention-to-treat analyses were applied for all inference analyses. When a biomarker distribution was not normally distributed, a natural-log transformation was performed. For bivariate statistics, we applied Wilcoxon’s (Mann-Whitney) test. Generalized linear regression modeling was applied for multivariate analyses of difference-in-difference effects of eggs on nutrient biomarkers. Low outliers were truncated to the value of the 2.5th percentile and high outliers were truncated to the value of the 97.5th percentile. Unadjusted and adjusted models included the corresponding baseline nutrient biomarker to avoid type I error (22). Adjusted models additionally adjusted for age of the child, sex of the child, and stunting status at baseline. The Benjamini-Hochberg method was used to adjust for multiple comparisons (23). Data analyses were performed with STATA software (version 13.1; StataCorp) and SAS software (version 9.4; SAS Analytics).

## RESULTS

**FIGURE 1:**
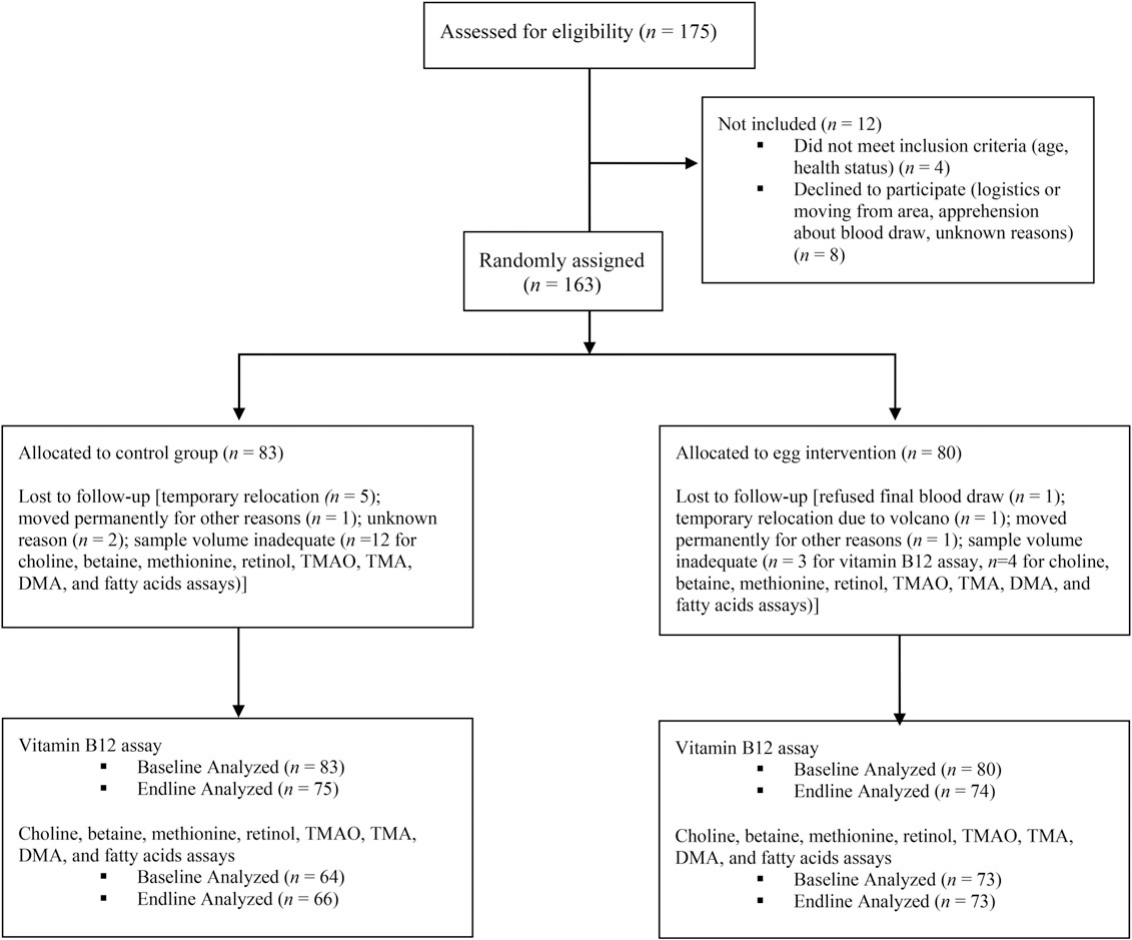
Flow diagram of participant progression through the randomized controlled trial. Infants aged 6–9 mo were randomly assigned to the treatment group (1 egg/d for 6 mo) or to a control group (no intervention). Socioeconomic data, anthropometric measures, and blood samples were collected at baseline and at endline, and weekly household visits were made for morbidity surveillance. Vitamin B-12 plasma concentrations were tested by using chemiluminescent competitive immunoassay; plasma concentrations of choline, betaine, dimethylglycine, methionine, retinol, linoleic acid, α-linolenic acid, DHA, TMAO, TMA, and DMA were tested by using LC-MS/MS. DMA, dimethylamine; LC-MS/MS, liquid chromatography–tandem mass spectrometry; TMA, trimethylamine; TMAO, trimethylamine-*N*-oxide.

Of the 175 mother (caregiver)-infant pairs assessed for eligibility, 4 did not meet the inclusion criteria and 8 declined to participate due to blood draw concerns, logistical challenges, or unknown reasons (12). A total of 163 infants were randomly assigned to the control (*n* = 83) and intervention (*n* = 80) groups (**Figure 1**). We were not able to collect a sufficient quantity of blood for all children at both time points. The numbers differed slightly due to the aliquot procedure that occurred in the field, filling first the tubes for the vitamin B-12 assay for analysis at NETLAB, followed by tubes for analyses with LC-MS/MS at the Washington University Metabolomics Facility, including all other biomarkers from this study.

Baseline socioeconomic factors in the children included in this biomarker analyses were comparable. As reported previously, baseline stunting, underweight, and wasting prevalence was significantly higher in the intervention group (12). Due to the high correlation among these markers, we selected only baseline stunting, which affected a higher proportion of children and was a primary outcome in the study, to include in all of the adjusted regression models.

The eggs used in the study, on average, provided >50% of daily infant (ages 8–13 mo) Recommended Dietary Allowance requirements for protein, vitamin B-12, and selenium (**Table 1**) (24). The choline content of individual eggs exceeded infant requirements by 29%. Comparisons of the study eggs with reported nutrient-composition values for eggs in the USDA database showed many similarities, with a few notable differences (25). Eggs reported in the USDA database were more highly concentrated in vitamins A and B-12 and folate than those used in the study, whereas concentrations of betaine, vitamin E, and calcium were higher in the study eggs.

**TABLE 1:** Nutrient composition of Lulun Project and USDA eggs^1^

			Ecuador Lulun Project eggs (whole, hard-boiled)^3^		
Nutrients	Unit	RDA/AI (7–12 mo)^2^	Per 52 g	Per 100 g	USDA eggs (whole, hard-boiled; per 100 g)^4^
Energy	kcal	769–858	81	158.82	155
Protein	g	11	6.8	13.33	12.58
Lipids (total)	g	30	5.93	11.63	10.61
LA	g	4.6	0.71	1.39	1.19
ALA	g	0.5	0.02	0.04	0.04
DHA	g	—	0.03	0.06	0.04
Carbohydrates	g	95	0.57	1.12	1.12
Vitamin A, RAE	μg	500	54.5	106.86	149
Vitamin B-12	μg	0.5	0.33	0.65	1.11
Folate, DFE	μg	80	15.1	29.61	44
Choline	mg	150	143.6	281.63	293.8
Betaine	mg	—	3.16	4.8	0.6
Vitamin E (α-tocopherol)	mg	5	1.9	3.73	1.03
Calcium	mg	260	30	58.82	50
Iron	mg	11	1.0	1.96	1.75
Magnesium	mg	75	6	11.76	10
Phosphorus	mg	275	100	196.08	178
Potassium	mg	700	68	133.33	126
Selenium	μg	20	19	37.25	30.7
Sodium	mg	370	70	137.25	124
Zinc	mg	3	0.58	1.14	1.05

1 AI, Adequate Intake; ALA, α-linolenic acid; DFE, dietary folate equivalents; LA, linoleic acid; RAE, retinol activity equivalents; RDA, Recommended Dietary Allowance.

2 RDA estimates for protein, iron, and zinc meet 97–98% of needs for infants aged 7–12 mo; AIs for energy and the remaining nutrients are mean intakes estimated from data for healthy breastfed infants from reference 24.

3 The nutrient content of Lulun Project eggs was tested by the Eurofins Scientific Nutrition Analysis Center (Des Moines, Iowa) in April 2016.

4 The nutrient content of USDA eggs was obtained from the USDA, Agricultural Research Service, Nutrient Data Laboratory (25).

Plasma concentrations of biomarkers did not differ by study group at baseline (**Table 2**). There was a general trend for decreasing choline concentrations from baseline to endline, which was buffered by the egg intervention. In addition, all of the essential fatty acids showed decreasing trends with age. At baseline, 11.4% of infants were vitamin B-12 deficient (<150 pmol/L) and 31.7% were marginally deficient (150–220 pmol/L). The prevalence of vitamin B-12 deficiency increased for both groups by endline: 15.4% of children were vitamin B-12 deficient and 43.6% of children were marginally deficient. No children were found to be vitamin A deficient (retinol concentration <0.7 μmol/L) at baseline or at endline, and only a small fraction were marginally deficient (retinol concentration <1.05 μmol/L) at baseline (3.1%) and at endline (0.7%).

**TABLE 2:** Effects of egg intervention on plasma concentrations of biomarkers^1^

	Plasma concentration of biomarkers, by study group^2^				Effect size difference between egg intervention group and control group					
	Baseline		Endline		Unadjusted^3^			Adjusted^4^		
	Control (*n* = 64)	Eggs (*n* = 73)	Control (*n* = 66)	Eggs (*n* = 73)	Difference (95% CI)	*P*	BH-*P*^5^	Difference (95% CI)	*P*	BH-*P*^5^
Choline, μg/mL	2.15 (2.06, 2.25)	2.05 (1.96, 2.14)	1.77 (1.69, 1.86)	2.00 (1.91, 2.10)	0.34 (0.11, 0.55)	0.004	0.032	0.35 (0.12, 0.57)	0.004	0.032
Betaine, μg/mL	8.70 (8.12, 9.33)	8.62 (8.13, 9.14)	8.30 (7.76, 8.87)	8.83 (8.25, 9.45)	0.29 (0.01, 0.56)	0.041	0.082	0.29 (0.01, 0.58)	0.043	0.086
DMG, μg/mL	1.10 (0.98, 1.23)	1.28 (1.17, 1.40)	0.80 (0.72, 0.90)	0.92 (0.85, 1.00)	0.03 (−0.20, 0.27)	0.769	0.772	0.03 (−0.22, 0.28)	0.785	0.785
Methionine, μg/mL	3.40 (3.24, 3.56)	3.16 (3.00, 3.34)	3.26 (3.05, 3.49)	3.61 (3.33, 3.91)	0.29 (0.00, 0.58)	0.047	0.165	0.31 (0.03, 0.60)	0.034	0.143
Vitamin B-12,^6^ pmol/L	279.10 (254.17, 306.48)	282.75 (255.18, 312.29)	250.44 (227.37, 275.85)	264.19 (243.52, 291.39)	0.08 (−0.17, 0.32)	0.576	0.772	0.12 (−0.16, 0.38)	0.335	0.536
Retinol, ng/mL	540.33 (508.38, 572.29)	531.82 (501.27, 562.37)	643.79 (601.18, 686.40)	656.35 (616.93, 695.76)	0.07 (−0.22, 0.38)	0.606	0.772	0.12 (−0.19, 0.42)	0.429	0.572
LA, μg/mL	14.42 (12.98, 16.01)	14.83 (13.51, 16.27)	12.53 (11.08, 14.17)	13.78 (11.87, 16.00)	0.23 (−0.05, 0.50)	0.108	0.162	0.23 (−0.06, 0.52)	0.114	0.171
ALA, μg/mL	1.06 (0.94, 1.20)	1.03 (0.92, 1.15)	0.89 (0.77, 1.04)	0.91 (0.76, 1.10)	0.10 (−0.17, 0.38)	0.459	0.459	0.07 (−0.21, 0.36)	0.610	0.610
DHA, μg/mL	1.37 (1.27, 1.49)	1.45 (1.32, 1.58)	1.14 (1.02, 1.26)	1.32 (1.15, 1.53)	0.42 (0.13, 0.71)	0.005	0.015	0.43 (0.13, 0.73)	0.006	0.018
TMAO, ng/mL	118.6 (91.3, 145.9)	126.5 (88.0, 165.0)	196.3(151.7, 240.9)	313.2 (221.2, 405.3)	0.33 (0.07,0.58)	0.013	0.034	0.33 (0.08, 0.58)	0.011	0.044
TMA, ng/mL	207.0 (176.7, 242.5)	222.0 (189.3, 260.4)	217.1 (178.5, 264.2)	190.1 (145.2, 248.8)	−0.04 (−0.40, 0.26)	0.772	0.772	−0.04 (−0.40, 0.26)	0.740	0.785
DMA, ng/mL	64.03 (59.22, 69.23)	68.36 (63.20, 73.94)	66.29 (60.54, 72.59)	78.95 (71.82, 86.77)	0.40 (0.11, 0.71)	0.009	0.034	0.37 (0.08, 0.69)	0.017	0.045

1 Results with the use of generalized linear regression modeling for unadjusted and adjusted effect sizes, by group, are shown. Effect size = mean difference/SD of the pooled data. ALA, α-linolenic acid; BH-*P*, Benjamini-Hochberg *P* value; DMA, dimethylamine; DMG, dimethylglycine; LA, linoleic acid; TMA, trimethylamine; TMAO, trimethylamine-*N*-oxide.

2 Values for methionine, TMAO, TMA, DMA, vitamin B-12, DHA, ALA, LA, betaine, choline, and DMG are geometric means (95% CIs); data were skewed. Values for retinol are means (95% CIs). All biomarker values are geometric means converted to log-normal distributions, except for retinol concentrations, which are presented as means.

3 Adjusted for corresponding baseline biomarker value.

4 Adjusted for child age, child sex, baseline stunting status, and corresponding baseline biomarker value.

5 Adjusted to account for multiple comparisons.

6 Available aliquots for vitamin B-12 analyses were for *n* = 81 and 78 in the baseline control and egg groups, respectively, and for *n* = 72 and 74 in the endline control and egg groups, respectively.

Although decreases in concentrations of some nutrients were observed longitudinally for children across groups, the egg intervention reduced this effect for choline and DHA. In adjusted regression modeling, the egg intervention significantly increased plasma concentrations of choline, betaine, methionine, DHA, TMAO, and DMA compared with those in the control group (Table 2). After adjustment for multiple comparisons, choline, DHA, TMAO, and DMA remained significant. No group effect was found for DMG, vitamin B-12, retinol, LA, ALA, TMA, or the ratios of betaine to choline and LA to ALA.

## DISCUSSION

In this 6-mo intervention trial in which 1 egg was provided daily early in the complementary feeding period, we found effects on choline and other biomarkers suggestive of effects in the choline methyl metabolism pathway. Concentrations of choline at endline were 2.00 and 1.78 μg/mL in the egg and control groups, respectively, which translated into an effect size of 0.35 when controlling for baseline choline, child age, sex, and stunting status. DHA concentrations at endline were 1.32 and 1.14 μg/mL in the egg and control groups, respectively. This corresponded to an adjusted effect size of 0.43 between groups. Although no effect on vitamin B-12 was detected from the egg intervention, 43.1% of the children were found to be marginal or deficient in the vitamin by the end of the trial.

We hypothesized an effect on choline nutrition, on the basis of the relatively high content of the nutrient found in eggs than in other foods (1). Choline is necessary for several critical pathways in growth and development, including the conversion to acetylcholine, phosphatidylcholine, and sphingomyelin. In this analysis, we focused on the one-carbon metabolism cycle, including betaine, DMG, methionine, and vitamin B-12 (26). This pathway is ultimately important for gene expression, because betaine converts to DMG, enabling the production of methionine from homocysteine and *S*-adenosylmethionine, which, in turn, donate methyl groups to methylate DNA (27). Our findings for significant increases in choline, betaine, and methionine concentrations in the egg group compared with the control group suggest that these pathways were affected either through changes in ratios or increased concentrations of the metabolites. There may have been an effect on concentrations of homocysteine in this pathway as well, but this metabolite was not measured. Methionine, an essential amino acid, is important during early development due to high demands for transmethylation and protein synthesis (28). Previous studies have shown a positive correlation between these biomarkers in young children (29). Interestingly, we observed no change in the betaine-to-choline ratio by group, which suggests a possible regulation of the proportionality of these metabolites regardless of intake or plasma concentration.

Recent attention has focused on the potential contribution of choline to linear growth promotion. One observational study showed that higher concentrations of serum choline were associated with a lower prevalence of stunting among young children aged 12–59 mo in Malawi (30). We previously reported that the egg intervention in the Lulun Project had a significant effect on increasing linear growth by a length-for-age *z* score of 0.63 and reducing stunting by 47% (12). However, our analyses showed only a partial mediating effect by choline. There may be contextual factors such as differences in the overall diet that influence the contribution of choline to growth. In our view, it likely reflects the importance of multiple other nutrients and bioactive factors converging to influence linear growth during infancy.

We found that there was a significant increase in TMAO concentrations in the egg intervention group, which may be related to choline nutrition. Choline is converted by the gut microbes into TMA, which is then absorbed and converted into TMAO in the liver (2). TMAO can be converted into DMA and is excreted in urine. A number of studies have reported postprandial increases in plasma TMAO concentrations after the ingestion of eggs (31–33), although one study reported no increase in fasting TMAO concentrations (34). TMAO has been implicated as a risk factor for the development of atherosclerosis and cardiovascular disease in adults (34); however, it is unclear whether elevated TMAO concentrations are of concern in early childhood. In the study in Malawian children described above, stunted children had higher TMAO-to-choline ratios than did nonstunted children (30); however, this appeared to be due to their lower choline concentrations rather than due to a difference in TMAO concentrations.

We measured 3 essential fatty acids in this study and found that eggs significantly increased plasma concentrations of DHA compared with controls, with a relatively large effect size of 0.43. It is widely established that DHA is crucial in neurodevelopment and growth, yet is lacking in the diets of young children in resource-poor settings (35, 36). During pregnancy and early childhood, there is rapid accretion of DHA in the brain and retinal tissue (37), with clear evidence of the importance of DHA to neurogenesis, neurotransmission, myelination, and synaptic plasticity, among other functions (38). We identified only one intervention study from a low- and middle-income country that examined fatty acid biomarker outcomes (39). The study conducted in the Gambia tested supplementation with DHA and EPA of infants aged 3–9 mo and found increased plasma n–3 fatty acid concentrations and midupper arm circumference at 9 mo, but no other growth or development effects. This trial in the Gambia showed higher DHA concentrations at baseline than our population of infants, and a small effect with supplementation, which investigators attributed to the high breast-milk concentrations of DHA in the population (39). The difference in DHA concentrations compared with our values may also be due to the metabolite measured; in the Gambia study, it was total DHA including free DHA and esterified DHA, whereas our values reflect only free DHA. To our knowledge, there are no validated reference ranges for plasma concentrations of DHA or other fatty acids in healthy populations of infants and young children to enable comparison with the Lulun Project children (40).

In this study, we also examined the change in median ratios for LA to ALA, which are important for conversion to long-chain fatty acids, and found no effect from the intervention. This effect, however, was not anticipated given the high ratio of n–6 to n–3 fatty acids in eggs compared with other foods (4). Boiled eggs (Table 1) contain DHA concentrations comparable to poultry meat (0.04 g/100 g), although higher than in animal milk or beef, but less than in fish such as herring (1.10 g/100 g) (25). The significant increase in DHA in the egg group could explain the growth effect we observed in this trial and potentially signify important contributions to brain development.

The prevalence of marginal or deficient vitamin B-12 at baseline among the Lulun Project infants (43.1%) was comparable to that in a sample of children aged 6–30 mo in India, but lower than in Guatemalan infants aged 12 mo (49%) (20, 21). We hypothesized that eggs, as an animal-source food, would improve vitamin B-12 status of infants in the intervention group. However, we observed no effect of the intervention on vitamin B-12 concentrations. Vitamin B-12 stores in both the children and breastfeeding mothers may have been sufficiently depleted to preclude improvement over 6 mo (41). Eggs have been shown to have lower bioavailability of vitamin B-12 relative to other animal-source foods (42). The prevalence of vitamin A deficiency was negligible. The national survey, ENSANUT-ECU, found 28.5% of children aged 6–11 mo and 16.2% of children aged 12–23 mo to be vitamin A deficient (serum retinol <20 μg/dL) (14). We did not hypothesize an effect of eggs on vitamin A because the content of vitamin A retinol activity equivalents in eggs is only ∼11% of the Dietary Reference Intake for this age group (Table 1).

Some limitations are present in this study. We were initially restricted with regard to the breadth of biomarkers explored due to funding limitations. To characterize the pathways affected by egg nutrition more fully, additional metabolites may be required, including amino acids, phosphatidylcholines, and other micronutrients, as well as bioactive compounds, such as insulin-like growth factor I (IGF-I). We may have also been limited by the sample size. In addition, there may have been limitations in the testing of the Lulun eggs for nutrient concentrations. Nutrient values were mostly comparable to USDA-tested egg concentrations, but there were some differences. For example, betaine concentration was markedly higher in the Lulun eggs. This may have been due to differences in poultry feed or possibly due to limitations in the long transit time to testing the eggs in the Iowa-based laboratory.

To our knowledge, this is the first study to examine the effect of an egg intervention early in the complementary feeding period on nutrition biomarkers, specifically choline and related metabolites, through an RCT. We hypothesized that the egg food matrix would deliver essential nutrients and other factors in a bioavailable manner. The Lulun Project showed important findings for increased linear growth and reduced stunting in young children, offering a unique opportunity to understand potential metabolic pathways that contribute to these effects. The egg intervention significantly increased choline, betaine, methionine, TMAO, DMA, and DHA concentrations. More exploration is needed to better elucidate the multiple other pathways that contribute to healthy growth and development of young children in resource-poor settings.
